# Low and no alcohol availability and sales in small retailers in Great Britain: A geographic longitudinal analysis from 2018 to 2022

**DOI:** 10.1111/add.70391

**Published:** 2026-03-19

**Authors:** Roberto Valiente, Helena Tunstall, Luke B. Wilson, Duncan Gillespie, Jamie Pearce, Niamh K. Shortt

**Affiliations:** ^1^ Centre for Research on Society and Health (CRESH), School of GeoSciences University of Edinburgh Edinburgh UK; ^2^ SPECTRUM Consortium UK; ^3^ Department of Geology, Geography and Environment (Geography Teaching Unit) University of Alcalá Alcalá de Henares Spain; ^4^ Public Health and Epidemiology Research Group, School of Medicine University of Alcalá Alcalá de Henares Spain; ^5^ School of Social and Political Science University of Edinburgh Edinburgh UK; ^6^ Sheffield Addictions Research Group (SARG), Sheffield Centre for Health and Related Research (SCHARR), School of Medicine and Population Health The University of Sheffield Sheffield UK

**Keywords:** alcohol availability, alcohol sales, commercial determinants of health, geographic inequalities, No/Lo alcohol, retail‐panel data, socio‐economic disparities

## Abstract

**Background and aims:**

The United Kingdom Government is committed to reducing alcohol consumption through increasing the availability of alcohol‐free and low‐alcohol (No/Lo) drinks; however, little is known about whether these products are equally available across different types of neighbourhoods, which may have implications for inequalities in potential health benefits or harms from exposure to No/Lo drinks. This study measured differences in the availability and sales of No/Lo products in small retailers across disparate types of neighbourhoods in Great Britain and over time.

**Design:**

A longitudinal geographic design using retail transaction data collected over 20 weeks seasonally distributed between 2018 and 2022.

**Setting:**

The study was conducted in Great Britain (England, Scotland and Wales).

**Participants/cases:**

11 278 479 alcohol transactions across 1432 small retailers in neighbourhoods with varying levels of socioeconomic deprivation and urbanicity.

**Measurements:**

No/Lo products were defined as alcoholic‐mimic beverages containing ≤1.2% alcohol by volume (ABV). Each week, we calculated retail‐level outcomes measuring No/Lo product availability defined as product range and sales volume (standardised as the number of serving units). Zero‐inflated Poisson regression models were used to assess differences in these outcomes by neighbourhood income deprivation and urbanicity over time.

**Findings:**

No/Lo sales volume tripled over the study period yet accounted for only 0.25% of total alcohol sales by 2022. In 2018, 34% of retailers reported sales of No/Lo products, rising to 68% by 2022. Retailers in low‐deprivation areas were more likely to sell No/Lo products and sold a wider product range compared with those in high‐deprivation areas ([incidence rate ratio (IRR) = 2.30, 95% confidence interval (CI) = 1.60–3.30 in 2022). No/Lo alcohol sales volume was statistically significantly higher among retailers in the least deprived neighbourhoods (IRR = 1.33, 95% CI = 1.14–1.57 in 2022) and rural areas compared with high‐deprivation and urban areas, but only in the most recent years.

**Conclusion:**

Alcohol‐free and low alcohol (No/Lo) product availability and sales increased among small retailers in Great Britain between 2018 and 2022, but these gains have been uneven, with greater access and uptake in more affluent and rural areas. This suggests emerging geographic disparities in access to and sales of No/Lo alternatives and their potential benefits or harms.

## INTRODUCTION

Over the past decade there has been a significant expansion in the availability and sales of no and low (No/Lo) alcohol drinks in high‐income countries [1], reflecting changes in consumer preferences and drinking behaviours. No/Lo beverages are alcohol‐mimicking drinks that contain no or low amounts of alcohol. Although there is no universal consensus on the alcohol‐by‐volume (ABV) threshold used to distinguish No/Lo products from standard alcoholic beverages, one commonly used definition is that adopted by the UK Government, which classifies No/Lo products as those containing 1.2% ABV or less [2]. A recent report analysing the No/Lo alcohol market across 10 countries (including the USA, France, Spain, Australia and Japan) documented annual growth rates exceeding 7% in all cases for the period 2018–2022, with a 15% annual increase in the UK. This growth is projected to continue at least until 2028 [3].

These trends are partially motivated by an increasing prioritisation of health and wellness, particularly since the COVID‐19 period. Drinks companies, including large transnational alcohol companies and niche start‐ups, have expanded the range and taste of their portfolio of No/Lo products [4]. These products are now more frequently encountered not only in supermarkets or convenience stores but also in bars and social venues [5].

Although the No/Lo alcohol market represents a relatively small share of the overall drinks market (below 2% in all countries examined) [3], the range of available products has expanded substantially to include beers, cider, wines, spirits and ready‐to‐drink cocktails (RTDs) [4]. In the UK, No/Lo alcohol sales more than doubled between 2018 and 2023, representing a cumulative volume growth of 88.6% [6].

Alcohol consumption is a well‐established risk factor for substantial health and social harms, including mortality, hospitalisations for non‐communicable diseases (such as cancers and heart disease), accidents, crime and violence [7]. Against this backdrop, No/Lo beverages represent not only a growing market segment but also a potential public health opportunity. By providing alternatives to standard alcoholic drinks, No/Lo products have the potential to reduce overall alcohol intake, a priority underscored in public health policies by entities such as the World Health Organization and public health agencies in the UK [8, 9]. However, evidence on whether No/Lo products serve as a substitute for alcoholic drinks or are consumed in addition to higher‐strength alcohol products remains equivocal [10].

A survey‐based study in the USA suggested that No/Lo consumption could potentially serve as a harm reduction strategy for individuals with alcohol use disorder [11], while two UK studies analysing sales data concluded that sales of no‐ and low‐alcohol beers replaced rather than added to sales of higher‐strength beers [12, 13]. In contrast, a study of Dutch university students suggests that most of this cohort were consuming alcohol‐free beverages in addition to their normal levels of alcohol consumption, with just a small group using them as a substitute [14]. These mixed findings—likely driven by heterogeneity in No/Lo product definitions, analytical approaches, and the disparate drinking patterns and preferences of study populations—underscore the need for further research on how No/Lo product sales and consumption vary across different populations and regions, and how they compare with those of standard alcoholic beverages.

In this context, it is important to consider who is consuming No/Lo products. Evidence suggests that these products are less popular among heavy drinkers [1], and emerging data indicate social gradients in No/Lo consumption by age, gender and socio‐economic status (SES) [4], with higher consumption observed among more affluent groups in the UK [15, 16, 17], Finland [18] and the USA [11]. This is particularly relevant given the substantial evidence that alcohol‐related harms are not evenly distributed: morbidity and mortality are higher among disadvantaged groups, despite similar or lower average alcohol consumption compared with more advantaged populations [19]. Identifying the pathways through which No/Lo beverages could function as substitutes for alcoholic drinks and reach populations at highest risk of alcohol‐related harms (i.e. heavy drinkers and less affluent communities) is therefore a key question that warrants further investigation.

One potential pathway is the availability and ease of access to No/Lo products, which may be a key factor influencing consumption. Research has begun to explore purchasing patterns for No/Lo products, with the results of an online experiment showing an increase in the purchase of No/Lo beverages when their availability was expanded [20]. The authors hypothesised that increased availability makes it easier for consumers to identify alternatives to alcohol as well as shifting expectations and norms around seeing and purchasing non‐alcoholic beverages.

To date, research has explored the availability of No/Lo alternatives at country level across the EU [21], as well as in bars and pubs in the UK [22] and in Australia [23]. To the best of our knowledge there is no comparable data on the availability of No/Lo products in neighbourhood stores, where alcohol beverages are typically offered as convenience goods for quick and spontaneous access, and often during long operating hours [24]. These local stores constitute an important source for alcohol provision, specifically for home alcohol consumption. Given that No/Lo consumption is reported to occur most commonly in the home environment [4, 11, 16], an improved understanding of No/Lo retail availability in local stores can help clarify the potential customer reach of these products.

The UK government committed to ‘deliver a significant increase in the availability of alcohol‐free and low‐alcohol’ drinks [9]. However, there is little evidence regarding differences in No/Lo sales and availability across neighbourhood type, including by area‐level deprivation. If, as suggested by this government commitment, No/Lo products have the potential to reduce harm, then any unequal distribution of these products may limit their harm reduction potential. On the other hand, if, as suggested by some authors, these products lead to the further normalisation of alcohol consumption [25], particularly among previously non‐drinking groups (e.g. children and young people) [26], then their unequal availability may reinforce existing inequalities.

High costs and the confidential nature of commercial transaction data have limited research exploring the spatial distribution of the availability and sales of No/Lo products, especially at the neighbourhood level, highlighting the need for further investigation. Using transaction‐level data, this article explores the availability and sale of No/Lo alcohol products in small convenience stores across Great Britain between 2018 and 2022. Based on previous work, we interpret the absences of No/Lo sales within retailers as ‘non‐availability’ [27, 28]. Using time series data, we assess the extent to which No/Lo availability and sales varied by neighbourhood deprivation and by urbanicity.

## METHODS

### Study design

This study employs a longitudinal design exploring the availability and sales of No/Lo alcohol in small conveniences stores across Great Britain between 2018 and 2022. Analyses were conducted in three phases. First, alcohol sales transaction data were obtained from point‐of‐sale electronic records for a sample of retailers. Next, we identified No/Lo alcohol sales and defined two outcome measures: (i) the range of different No/Lo products sold; and (ii) sales volume, measured in standard serving units. Finally, we ran regression models to assess how these outcomes varied across neighbourhoods with different levels of income deprivation and urbanicity (independent variables). The models were based on a series of hypotheses derived from existing evidence. We hypothesised that No/Lo availability and sales are higher among retailers in less deprived and more urban areas. We also anticipated that No/Lo availability and sales have increased over time since 2018.

### Alcohol and No/Lo sales data

The Retail Data Partnership (TRDP), a company that supplies electronic point‐of‐sale tills (https://shopmate.co.uk), provided alcohol sales records (both No/Lo and standard alcohol) from all convenience stores that used their system in Great Britain. The convenience stores in this study, were small‐scale retail establishments that offer a limited selection of frequently purchased items—such as snacks, beverages (including alcohol), tobacco products, basic groceries and household essentials—with extended opening hours and an emphasis on speed and convenience, rather than price or product variety. These stores operate either independently or within franchise or symbol group systems (e.g. Premier, Londis, Costcutter). We were unable to assess sales records from Northern Ireland, to provide a complete view of UK patterns, owing to the low number of retailers listed in the TRDP data set. To account for potential seasonality in alcohol sales, data were sampled from 1‐week periods (7th–13th) in March, June, September and December of each year between 2018 and 2022, totalling 20 weeks. One‐week periods allow us to avoid potential differences between weekday and weekend sales. These months were chosen to avoid major holiday periods that are known to substantially influence alcohol purchasing behaviour, such as summer, Christmas and Easter (typically occurring in April) holiday periods. This time frame was chosen to capture any changes related to the COVID‐19 pandemic (representing pre‐, during and post‐COVID‐19 sales).

Sales records were defined as each item scanned on the till within each basket. Each record contained information on the name of the alcohol product, the EAN‐13 (13‐digit) barcode, pack size (mL), the product type within a range of alcohol product categories (beer, cider, RTD, also known as ‘alcopops’, spirits and wine), the alcohol strength (ABV), the date and time of purchase, and an identifier of the Lower‐layer Super Output Area (LSOA) or Data Zones (DZs) where the retailer operates. In the UK, both LSOAs and DZs are geographic areas used for statistical reporting. LSOAs are area units operating in England and Wales, containing roughly 1000–3000 residents. DZs are area units used in Scotland, representing roughly 500–1000 inhabitants [29].

We obtained a total of 11 679 856 alcohol sales records from a longitudinal cohort of 1440 retailers within the study area and period. All retailers included in the cohort operated for at least 5 days each week and for at least 2 hours on each day it was open, to filter for retailers with regular activity across the study period. These criteria have been used in previous research [30]. From this sample, we removed all sales records with missing data on the ‘alcohol product type’, ‘alcohol strength ABV’ and ‘pack size’ variables. Additionally, data from retailers where transactions with missing issues, as described above, exceeded 40% of their total sales records were removed. In the remaining retailers, the median percentage of transactions with missing issues was 0.44% (IQR = 0.11%–2.37%). This cleaning procedure yielded a total of 11 278 479 sales records from 1432 retailers.

We used the UK Government alcohol definition to distinguish between No/Lo alcohol products (those with 1.2% ABV or less) and standard alcohol products (alcohol beverages with more than 1.2% ABV) [2].

### Outcome variables

We examined two distinct but inter‐related outcome variables to capture multiple dimensions of the availability and sales of No/Lo alcohol products at the retailer level: product range and sales volume.

#### No/Lo alcohol product range

We measured the number of unique No/Lo barcodes sold per retailer to describe the range of products sold per retailer and week. This measure was used as a proxy of No/Lo product availability by capturing the diversity of options. It is important to note that we did not have information on the full range of stock stored at each shop, our data only described recorded sales, so we used sales data as a proxy variable for the number of different products stocked by the retailers. Previous research exploring retailer product ranges using detailed store‐level scanner data has taken a similar approach when assessing store inventories and availability [27, 28].

#### No/Lo alcohol sales volume

To complement the product range indicator, we calculated a second variable to capture the volume of product sold. Measuring alcohol volume for No/Lo products is challenging, as standard methods, like converting to pure alcohol or using the package size, can be misleading owing to variable serving sizes and alcohol content. To improve comparability, we used standard serving units as our metric. Servings were calculated by dividing the volume sold by a typical serving size for each beverage type, based on median volumes in our sales data, excluding products over 1 L to avoid any skewing from multi‐packs. We defined a standard serving as 500 mL for both No/Lo beers and ciders, and 330 mL for No/Lo RTD beverages. As wines and spirits are typically sold in bottles, we referred to experimental studies indicating that a self‐poured glass of wine is usually around 175 mL (a medium glass), and a self‐poured measure of spirits approximates 50 mL (a double serving) [31]. Finally, we aggregated weekly sales in serving units for each retailer and product type.

### Independent variables

We linked neighbourhood‐level indicators of income deprivation and urban/rural status to each retailer at the LSOA/DZ level. Income deprivation was derived from the income domain of the most recently available Indices of Multiple Deprivation: the English and Welsh indices from 2019, and the Scottish index from 2020. The income domains of these national indices are defined in similar ways based upon the proportion of the population receiving various forms of means‐tested benefits (e.g. Income Support, Tax Credits, Guaranteed Pension Credit) [32, 33, 34].

Using the most recent England and Wales (2021) and Scottish (2022) Government Urban–Rural classification, we classified retailers nested in settlements larger than 3000 inhabitants as urban; otherwise, we coded them as rural [35, 36].

### Statistical analysis

Statistical analyses were conducted in two stages. First, we described the proportion of retailers selling No/Lo products by levels of neighbourhood income deprivation, urbanicity and alcohol product type. These proportions were computed annually from 2018 to 2022 to examine trends over time. We also calculated both absolute and relative percentage changes between 2018 and 2022.

Second, we used regression models to assess the association between neighbourhood‐level deprivation and our two primary outcome variables: (i) the range of No/Lo products sold (product range); and (ii) the total volume of No/Lo alcohol sold, expressed in standard serving units (sales volume).

Given the characteristics of each outcome, we applied different modelling strategies. For sales volume, which included a high proportion of zero observations and for which modelling the likelihood of zero sales was substantially relevant to our objectives, we used zero‐inflated Poisson models. These allowed us to: (i) account for the high probability that a retailer does not sell No/Lo alcohol products (zero‐inflation component); and (ii) estimate the association between neighbourhood deprivation or urbanicity and the volume sold [37]. The negative binomial model was preferred for analysing product range, owing to a better performance compared with Poisson models and more complex zero‐inflated alternatives. Full diagnostic metrics for all models tested are presented in Table S1. Negative binomial models accounted better for overdispersion in the count data than other model specifications. Model fit was assessed using the Akaike information criterion (AIC), the Bayesian information criterion (BIC) and pseudo‐*R* ^2^ statistics.

We classified retailers into income deprivation tertiles (T1 = most deprived, T3 = least deprived) and urban/rural categories, calculated separately for each country using the national indices mentioned above. Sensitivity analyses using income deprivation quintiles produced similar results to the ones obtained with tertile classification, so tertiles were retained to balance interpretability, statistical power and model stability. Retailers were also classified by country, but this variable was excluded from the analysis because of the unequal distribution of outlets (England, *n* = 1094; Scotland, *n* = 139; Wales, *n* = 199), which limited comparability. Therefore, final multi‐variable models were adjusted for income deprivation and urbanicity. We also adjusted for standard alcohol sales (SAS) as a covariate, as retailers with higher SAS tended to sell greater volumes of No/Lo alcohol.

We additionally added years (2018–2022) as an interaction term in the fully adjusted models to explore how associations evolved over time. Furthermore, we ran stratified models by No/Lo alcohol type, focusing on the three most sold categories (beer, cider and wine), to investigate whether patterns varied by product category. Sensitivity analyses for standard alcoholic beverages were conducted using identical model specifications, differing only in the outcome variable, to provide contextual comparison with trends observed for No/Lo products.

Model estimates are reported as odds ratios (ORs) for the zero‐inflation component of the models, and as incidence rate ratios (IRRs) for the count component of the models, with 95% confidence intervals (95% CIs), comparing levels of deprivation and urbanicity relative to the most deprived and urban groups, respectively.

All analyses were exploratory (not pre‐specified in a protocol or statistical analysis plan), and conducted using the ‘stats’, ‘pscl’ and ‘MASS’ packages in R 4.4.1 (R Foundation for Statistical Computing, Vienna, Austria).

## RESULTS

### Trends in No/Lo alcohol product range and sales volume

Over the study period, there was a progressive increase in the proportion of retailers selling No/Lo alcohol products (Table 1). In 2018, only 34% of retailers sold No/Lo products, rising to 68% in 2022. This trend was observed across all types of neighbourhoods, although retailers located in less deprived areas consistently showed higher availability. We did not observe large differences in the proportion of retailers selling No/Lo products between urban and rural areas through time. The results suggested that the proportions seemed to be higher in England, but this value should be interpreted cautiously owing to the relatively low sample of retailers in Scotland and Wales. No/Lo beers were the most available product type, with almost 62% of retailers selling these products in 2022, followed by No/Lo ciders (23%) and wines (16%). The availability of these three No/Lo subtypes had increased by more than 200% since 2018. The proportion of retailers selling No/Lo RTDs and spirits experienced a limited absolute increase from 2018, reaching their peak in 2022. Nevertheless, their availability remained low, not exceeding 3% of retailers.

**TABLE 1:** Proportion of retailers selling No/Lo products by retailer type and year.

Area domain	Area type	*n*	2018	2019	2020	2021	2022	Absolute % change 2018–2022	Relative % change 2018–2022
Total	–	1432	34.3%	45.2%	60.1%	68.0%	67.9%	+33.6%	198.0%
Income deprivation	T1—high	653	29.7%	40.4%	55.0%	63.4%	62.8%	+33.1%	211.3%
	T2	488	38.3%	50.4%	64.8%	72.1%	72.1%	+33.8%	188.2%
	T3—low	291	37.8%	47.1%	63.6%	71.5%	72.2%	+34.4%	190.9%
Urbanicity	Urban	1053	33.6%	43.8%	61.3%	68.5%	68.1%	+34.4%	202.5%
	Rural	379	36.2%	49.1%	56.7%	66.8%	67.3%	+31.1%	186.1%
Nation	England	1094	37.4%	47.5%	63.3%	70.7%	71.3%	+33.9%	190.7%
	Scotland	139	36.0%	43.2%	48.9%	61.2%	54.7%	+18.7%	152.0%
	Wales	199	16.1%	33.7%	50.3%	58.3%	58.3%	+42.2%	362.5%
Total alcohol sales volume	T1—high	477	50.1%	63.3%	77.6%	84.7%	83.0%	+32.9%	165.7%
	T2	477	31.7%	44.2%	60.8%	71.9%	71.9%	+40.3%	227.1%
	T3—low	478	21.2%	28.0%	41.8%	47.5%	48.7%	+27.6%	230.7%
NoLo alcohol product type	Beer	1432	28.4%	39.0%	49.5%	61.9%	61.8%	+33,5%	218.0%
	Cider	1432	6.6%	14.9%	29.8%	27.7%	23.3%	+16,6%	350.7%
	RTDs	1432	0.2%	0.2%	0.3%	0.5%	0.9%	+0.7%	433.3%
	Spirit	1432	0.1%	0.2%	0.3%	2.2%	2.2%	+2.0%	1542.9%
	Wine	1432	6.8%	5.9%	8.0%	12.2%	15.9%	+9.2%	235.2%

Abbreviations: No/Lo = no and low alcohol; RTD = ready‐to‐drink cocktails.

**FIGURE 1:**
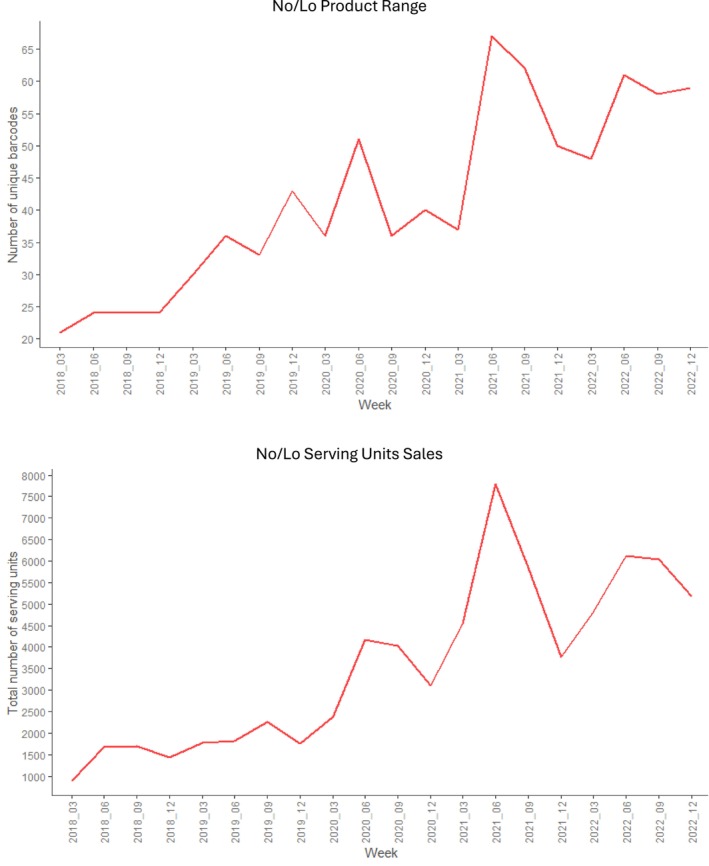
Total No/Lo alcohol availability by product range (number of unique barcodes, upper panel) and sales volume expressed in serving units (lower panel) per week.

Figure 1 presents weekly No/Lo alcohol availability over time by product range (upper panel) and by sales volume expressed in serving units (lower panel). Overall, both indicators exhibit a clear upward trend over time, with the most substantial growth occurring from 2020 onwards. The number of unique barcodes steadily increased, particularly between 2020 and 2021, suggesting an expansion in the variety of products sold by retailers. Sales volume also rose, particularly from 2020 onwards, showing clear seasonal patterns in 2020, 2021 and 2022, with higher sales during the summer weeks (June) compared with winter weeks (December). For context, trends in standard alcoholic beverage sales did not show a comparable sustained increase over the study period; temporary increases in sales were observed in 2020 and 2021, likely reflecting COVID‐19‐related disruption, while in 2022 the sales levels did not significantly differ from those observed in 2018–2019 (results not shown).

Despite the upward trend, No/Lo alcohol sales remained marginal in the overall alcohol market in Great Britain. As of December 2022, they accounted for just 0.25% of total alcohol sales within our retailer sample.

### Neighbourhood differences in No/Lo alcohol product range and sales

**FIGURE 2:**
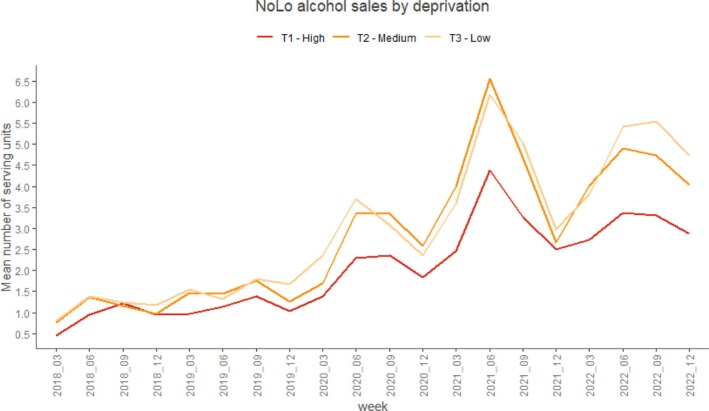
Mean weekly No/Lo alcohol sales volume by neighbourhood income deprivation.

Figure 2 shows the mean No/Lo alcohol sales volume across retailers in neighbourhoods with varying levels of deprivation. Retailers in the least deprived tertile sold more No/Lo alcohol serving units per week compared with those in the most deprived tertile. Descriptive analysis over the product range variable showed a very similar pattern: retailers in the least deprived areas sold a broader range of No/Lo products compared with retailers in the most deprived areas (Figure S1). Descriptive analyses showed no substantial differences in the mean number of No/Lo alcohol serving units or unique products sold across urban and rural retailers (Figure S2).

**FIGURE 3:**
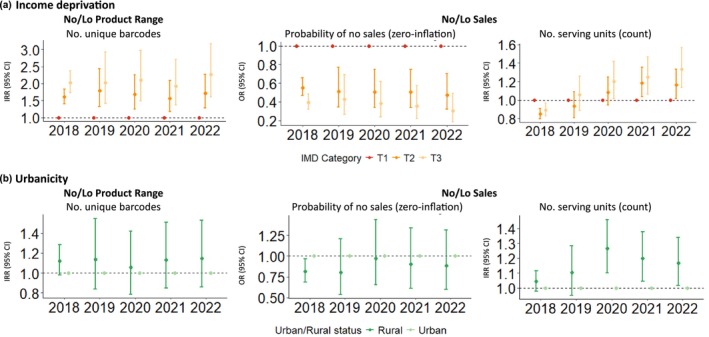
Association between No/Lo alcohol product range and sales volume and neighbourhood income deprivation and urbanicity across retailers, 2018–2022.

Figure 3 presents the results of the regression analyses examining the associations among neighbourhood income deprivation, urbanicity and the No/Lo alcohol outcomes. Results from the zero‐inflation component of the models, which estimates the likelihood of zero sales, showed that retailers located in the least deprived neighbourhoods were consistently less likely to report no sales of No/Lo alcohol compared with those in the most deprived areas, a pattern observed across all years (e.g. OR = 0.38 in 2018; OR = 0.30 in 2022). Retailers in more affluent areas also sold a wider range of No/Lo products, with differences that were consistent over time and appeared to grow larger (IRR = 1.91, 95% CI = 1.69–2.42, in 2018; IRR = 2.30, 95% CI = 1.60–3.30, in 2022). Similarly, No/Lo alcohol sales volume was significantly higher among retailers in the least deprived neighbourhoods. These differences became statistically significant from 2020 onwards and continued to widen over time (IRR = 1.20 in 2020; IRR = 1.33 in 2022). All these associations remained robust in both univariate models and after adjusting for urbanicity and SAS. For simplicity, only the latter models are presented in this article. Tables S2–S4 in the supporting information present the results of intermediate models.

Our models did not show statistically significant differences by No/Lo product range across urban or rural retailers. Univariate models show that rural retailers sold a higher number of No/Lo serving units as compared with those in urban areas (IRR = 1.08, 95% CI = 1.06–1.10). However, this association only remained significant for 2020, 2021 and 2022, after adjusting by neighbourhood income deprivation and retail‐level standard alcohol sales (Figure 3).

**FIGURE 4:**
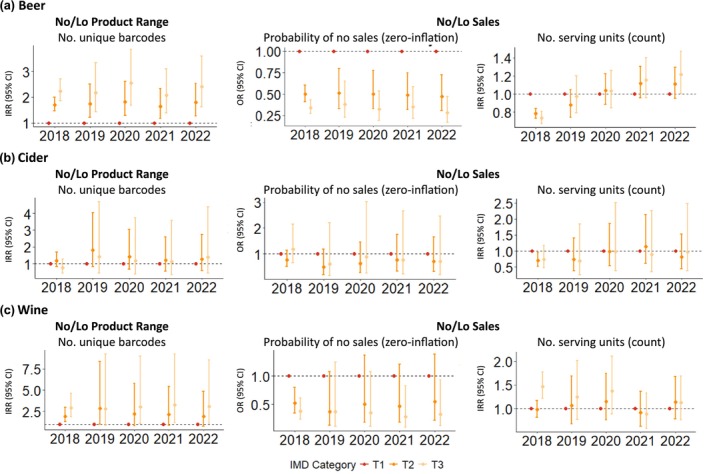
Association between No/Lo alcohol product range and sales volume and neighbourhood income deprivation across retailers stratified by No/Lo alcohol product type, 2018–2022.

Stratified analysis by product type is presented in Figure 4. We found significant differences in the No/Lo beer product range by neighbourhood deprivation. The range of No/Lo beer products sold was 2.00–2.55 times larger among retailers in the most affluent neighbourhoods (vs retailers in the most deprived neighbourhoods). The zero‐inflation component of the models indicated that retailers in the least deprived areas had 70% less chance of not selling No/Lo beers as their counterparts in the most deprived areas across all years. However, the count component of the models for sales volume (restricted to retailers selling No/Lo alcohol) only reported significant differences in No/Lo beer sales volume between the least and most deprived areas in 2022 (IRR = 1.22, 95% CI = 1.01–1.47). No statistically significant differences were observed for cider or wine in terms of product range and sales volume by neighbourhood deprivation. Similarly, no significant differences were found by urbanicity across any product type (Figure S3).

## DISCUSSION

### Summary of findings and interpretation

This study is the first to use retail transaction data to provide evidence on trends for the availability and sales of No/Lo alcohol products in small retail settings across Great Britain over multiple years. Our findings show that although No/Lo products have become more available and their sales have risen between 2018 and 2022, their market share remains marginal relative to overall alcohol sales.

Importantly, the growth in No/Lo product availability and sales has not been evenly distributed across neighbourhoods. We found that the availability and sales of No/Lo products were higher in less deprived areas. Specifically, we observed a significantly greater product range and greater sales in less deprived areas, particularly from 2020 onwards, alongside a clear widening of disparities over time. These findings align with previous research showing that No/Lo alcohol consumption is greater among higher income groups [11, 15, 16, 17, 18]. A similar pattern has been widely documented in the context of healthier foods, where access tends to be greater in higher‐income communities [38].

In contrast, we found no consistent differences by urbanicity, with only minor evidence that rural retailers sold slightly more No/Lo units in the later years. This does not fully support our expectation that urban areas would have greater No/Lo product range and sales, suggesting that urban–rural location may not emerge as a strong independent determinant. Its role may be shaped by or overlap with underlying socio‐economic differences, at least in small‐format retail contexts.

Lastly, product type analysis revealed notable patterns. No/Lo beer dominated the No/Lo market, while cider and wine showed more limited availability and sales, with no clear socio‐economic trends. No/Lo RTDs and spirits were rarely sold across small retailers, indicating that product diversity within the No/Lo segment remains limited and concentrated in a few beverage types. This finding aligns with previous research highlighting the challenges of adapting certain types of alcoholic beverages to No/Lo formats. Factors such as flavour, sensory profile, aroma, mouthfeel, colour, chemical composition and even sound have been identified as key influences on consumer willingness to try and adopt No/Lo alternatives [4].

### Implications for public health

Our findings suggest a geographical pattern of drinking preferences that can be interpreted through two complementary, but non‐exclusive, perspectives. On the demand side, socio‐economic differences in consumption preferences and purchasing power are likely to translate into spatial patterns of No/Lo alcohol sales, with higher uptake in areas where more affluent populations are concentrated. On the supply side, and consistent with the commercial determinants of the health framework, the influence of large transnational alcohol companies is also geographically patterned [39]. Product availability, range, and promotional intensity and advertising are shaped by market logic that often favours areas perceived as more commercially attractive, thereby reinforcing the geographically uneven exposure to No/Lo products. These dynamics suggest that public health responses to the expansion of No/Lo products should consider a geographical lens, accounting for regional contexts and inequalities.

While the evidence remains inconclusive as to whether No/Lo products provide a substitute for or add to overall alcohol consumption [10], a geographical perspective allows for several important observations to be made from our findings. Under the assumption that the increased availability of No/Lo products has the potential to contribute to alcohol harm reduction, our results suggest that any such benefits are currently accruing disproportionately within wealthier communities, raising concerns that the inequitable distribution of No/Lo products may contribute to widening inequalities in alcohol‐related harm. On the other hand, if it transpires that a key public health implication of the greater availability of No/Lo products is to increase the local normalisation of alcohol, then efforts to increase the availability in low‐income neighbourhoods may sustain or even exasperate alcohol‐related harms.

Assuming No/Lo products act as a substitute for alcohol, ensuring equitable availability and access to No/Lo alcohol alternatives could be a key consideration in public health planning. Potential policy responses might include incentives for retailers in more deprived areas to stock No/Lo options, or policy awareness campaigns promoting No/Lo consumption as an alternative to standard alcohol, tailored to diverse social groups. Previous studies have highlighted that No/Lo products are less likely to be available on price promotion and may be more expensive than their alcoholic counterparts [40]. Improving their affordability may therefore be crucial to facilitate access in more deprived communities and enhance their potential as alcohol substitutes.

Such measures could operate alongside established population‐level interventions such as alcohol taxation and minimum unit pricing (MUP). Alcohol taxes increase the price of standard alcoholic beverages, which may shift consumption towards non‐ or lower‐taxed alternatives. Maintaining No/Lo products within lower duty brackets—for example through lower or zero excise duty or preferential VAT treatment, compared with standard alcoholic beverages—could strengthen their substitution potential. Similarly, as MUP does not apply or applies minimally to No/Lo products, increases in the price of cheap alcohol may make No/Lo options relatively more attractive, particularly for price‐sensitive consumers.

However, while increased availability and sales of No/Lo products is often framed as a public health opportunity, this perspective requires careful scrutiny. Increasing the availability and sales of No/Lo products may contribute to the normalisation of alcohol use. If this is the case, then major producers of high‐strength alcohol are increasingly involved in the development and promotion of No/Lo products, often using them to enhance brand visibility and legitimacy, which could drive sales across their full portfolios, including higher‐alcohol beverages [4]. Such practices may normalise drinking behaviours and indirectly reinforce demand for higher‐strength products, thereby undermining anticipated public health benefits [41, 42, 43, 44]. Concerns have also been raised regarding a possible ‘gateway’ effect—where early exposure to alcohol‐like products may lower barriers to subsequent alcohol initiation—particularly among children and adolescents, who can legally purchase zero‐alcohol beverages and may struggle to distinguish them from alcoholic products owing to their near‐identical branding and packaging [26, 45, 46]. This raises questions about the role of No/Lo products in shaping drinking norms from an early age. Thus, from an alcohol normalisation perspective, incentivising the availability and sales of No/Lo beverages overall—and particularly in neighbourhoods with high levels of deprivation—may not be an effective measure, as it could inadvertently encourage alcohol consumption and thereby exacerbate inequalities. Future studies should address the extent to which these industry‐led practices influence consumer demand and behaviours, and how they may be contributing to the geographical and socio‐economic inequalities in No/Lo availability and sales, as observed in this study.

Although we found that No/Lo products represent a growing share of the alcohol market, our results do not allow us to determine whether No/Lo products displace or supplement alcohol consumption. As this study does not assess behavioural outcomes, causal effects related to alcohol substitution or assess the normalisation effect of No/Lo drinks exposure, both interpretations are plausible. However, the main contribution of our work is to show that the expansion of No/Lo availability and sales is socially and geographically patterned, with disproportionate growth in less deprived neighbourhoods over time. This means that any such potential benefits or harms are unlikely to be evenly realised under current market conditions. These findings highlight the need for further research to address the substitution versus addition question in No/Lo alcohol research, particularly by examining: (i) who is being reached by No/Lo products; (ii) which populations do not have access; and (iii) how retail availability may shape consumption opportunities across different geographies. Until stronger evidence is available on harm‐reduction effects, consumption patterns and potential unintended exposures, policy efforts to actively promote No/Lo availability should therefore be approached with caution.

### Strengths and limitations

This study has several strengths. While previous research has discussed socio‐economic inequalities in No/Lo alcohol consumption, most studies have relied on consumer surveys. To the best of our knowledge, no prior studies have quantified No/Lo alcohol product range and sales at the retail and neighbourhood levels. By using longitudinal and detailed point‐of‐sale data, our study provides a more robust, granular and geographically sensitive understanding of market dynamics than analyses based on self‐reported consumption.

Although this study focuses on the UK, it remains relevant internationally. The UK is among the 10 largest No/Lo alcohol markets globally [3], making it a key case study for anticipating trends in countries where the No/Lo market is less developed or alcohol regulation is weaker—contexts in which socio‐economic disparities may be even more pronounced.

It is important to acknowledge the limitations of this study. The TRDP data set is derived from an opportunity sample of retailers. According to a 2021 report, there were approximately 47 079 convenience stores in the UK, meaning our sample represents roughly 3% of the total [47]. Although the retailers in our sample were geographically distributed across the study area and the sample size provided sufficient power to detect differences in No/Lo alcohol availability and sales by neighbourhood deprivation and urbanicity, our analysis was limited to comparisons between different nations within Great Britain. This represents a structural aspect of the population distribution, as England accounts for the majority of the population in Great Britain (over 56 million, compared with approximately 5.5 million in Scotland and 3.1 million in Wales), and the distribution of retail infrastructure—including convenience stores—is therefore uneven across the nations [48]. Further, 73% of our sample of retailers were located in urban areas and 45% of them were in neighbourhoods categorised at the highest tertile of income deprivation. However, this skewed geographical distribution likely reflects the inherent spatial pattern of convenience store locations in the UK. For instance, it has been estimated that 63% of convenience stores are located in urban areas [47], and previous research has highlighted the higher density of convenience stores in the most deprived neighbourhoods (vs least deprived areas) [49].

While this did not limit comparisons on No/Lo availability and sales between urban and rural retailers, it did prevent analyses examining interactions between income deprivation and urbanicity, owing to limited representation in some deprivation subgroups among rural settings. Recognising the spatial patterning of retail environments is therefore important for understanding which communities and places are most likely to benefit from or be affected by the expansion of No/Lo products and related policy interventions.

Additionally, the data used in this study reflect sales rather than stock levels, which limits our ability to assess the full range of No/Lo products stocked by retailers. As a result, rarely purchased No/Lo products may be under‐represented in our analyses. We discuss ‘availability’ using sales data acting as a proxy for item availability. However, we acknowledge that some stores may stock No/Lo products with no sales during our sample weeks. Our data do, however, cover a substantial time period and it could be hypothesised that retailers will optimise their inventory by discontinuing items with low or no sales.

Turnover levels may also vary systematically across areas with different levels of deprivation and urbanicity. To address this limitation, we used a variable accounting the standard alcohol sales volume in our models to control the effect of total turnover on the No/Lo product range and sales outcomes. Further, as our analysis includes a spatial component, we acknowledge the potential for spatial correlation or non‐independence among observations, which could lead to pseudo‐replication and an overestimation of degrees of freedom. Although further modelling incorporating spatial random effects could help address this issue, only 2.5% of retailers in our sample were located in the same LSOA/DZ as another retailer, minimising the risk of spatial dependence.

Our study focused specifically on convenience stores, a specific type of off‐premises outlets. Data from other types of alcohol retailers, such as on‐premises venues or larger supermarkets, were not available. Nonetheless, previous research has highlighted the importance of exploring alcohol and No/Lo alcohol availability at off‐sale retailers, driven by the increasing trend of alcohol consumption in home environments [4, 11, 16]. Furthermore, off‐premises outlets often provide alcohol at lower prices and with a broader product range than on‐premises venues, making them a popular choice among consumers. While it is likely that No/Lo product availability and sales are higher in large supermarkets, convenience stores occupy a key position in the alcohol retail landscape owing to their accessibility, extended operating hours and suitability for spontaneous purchases. To our knowledge, this is the first study in Great Britain to specifically investigate No/Lo alcohol availability and sales in local convenience retailers. However, there are opportunities for further studies to explore the availability and sales of No/Lo alcohol products in larger retail settings, where the existing literature remains scarce and geographical disparities are not known.

A further limitation of this study is that the TRDP data only cover sales up to 2022 and do not capture more recent changes in No/Lo availability or sales. The market has continued to evolve rapidly, and while overall sales are likely to have increased further [3, 5], the impact on recent geographic patterns and socio‐economic disparities is unknown.

## CONCLUSION

No/Lo alcohol products are increasingly available in the British retail market but remain a small share of overall alcohol sales. This growth has been uneven, with more affluent neighbourhoods gaining faster and broader access to these products. Although evidence remains limited on whether No/Lo products reduce alcohol‐related harm, monitoring their evolution through a geographical equity lens is essential. Our findings suggest that any benefits—or harms—are unlikely to be evenly distributed under current market conditions. Stronger evidence is needed on the capacity of No/Lo products to substitute for alcohol, to reach individuals at highest risk of alcohol‐related harm, and on unintended exposures among vulnerable populations such as children and adolescents. Policy efforts to actively promote No/Lo availability should therefore be approached cautiously, accounting for regional contexts and existing geographic inequalities.

## AUTHOR CONTRIBUTIONS

**Roberto Valiente:** Conceptualization; investigation; writing—original draft; methodology; validation; visualization; writing—review and editing; software; formal analysis; project administration; resources. **Helena Tunstall:** Conceptualization; writing—review and editing; resources; methodology; validation; investigation. **Luke B. Wilson:** Methodology; validation; writing—review and editing; investigation; resources. **Duncan Gillespie:** Investigation; methodology; validation; writing—review and editing; supervision; resources. **Jamie Pearce:** Conceptualization; investigation; funding acquisition; writing—review and editing; methodology; validation; data curation; supervision; project administration. **Niamh K. Shortt:** Data curation; supervision; project administration; writing—review and editing; validation; methodology; investigation; funding acquisition; conceptualization.

## DECLARATION OF INTERESTS

The authors declare that they have no competing interests.

## Supporting information


**Figure S1.** Mean weekly No/Lo alcohol product range by neighbourhood deprivation.
**Figure S2.** Mean weekly No/Lo alcohol sales volume (upper panel) and product ranges (bottom panel) by neighbourhood urbanicity.
**Figure S3.** Association between No/Lo alcohol product ranges and sales volume and neighbourhood urban/rural status across retailers, stratified by No/Lo alcohol product type, 2018–2022.
**Table S1.** Model diagnostics for associations among No/Lo alcohol product range, sales volume and retailer‐level predictors, 2018–2022. (Observations, *n* = 28 346).
**Table S2.** Association between No/Lo alcohol product range and sales volume and neighbourhood income deprivation and urbanicity across retailers, 2018–2022. Univariate non‐adjusted models.
**Table S3.** Association between No/Lo alcohol product range and sales volume and neighbourhood income deprivation and urbanicity across retailers, 2018–2022. Intermediate models include neighbourhood income deprivation and urbanicity.
**Table S4.** Association between No/Lo alcohol product range and sales volume and neighbourhood income deprivation and urbanicity across retailers, 2018–2022. Intermediate models include neighbourhood income deprivation, urbanicity and standard alcohol sales.

## Data Availability

The data that support the findings of this study are available from the corresponding author upon reasonable request.
