# Supplementary material for: Low and no alcohol availability and sales in small retailers in Great Britain: A geographic longitudinal analysis from 2018 to 2022
Source: Addiction. 2026 Mar 19;121(7):1863–75. doi: 10.1111/add.70391 (PMC13291110; doi:10.1111/add.70391)
Supplement: Supplementary file 1 — Figure S1. Mean weekly No/Lo alcohol product range by neighbourhood deprivation. Figure S2. Mean weekly No/Lo alcohol sales volume (upper panel) and product ranges (bottom panel) by neighbourhood urbanicity. Figure S3. Association between No/Lo alcohol product ranges and sales volume and neighbourhood urban/rural status across retailers, stratified by No/Lo alcohol product type, 2018–2022. Table S1. Model diagnostics for associations among No/Lo alcohol product range, sales volume and retailer‐level predictors, 2018–2022. (Observations, n = 28 346). Table S2. Association between No/Lo alcohol product range and sales volume and neighbourhood income deprivation and urbanicity across retailers, 2018–2022. Univariate non‐adjusted models. Table S3. Association between No/Lo alcohol product range and sales volume and neighbourhood income deprivation and urbanicity across retailers, 2018–2022. Intermediate models include neighbourhood income deprivation and urbanicity. Table S4. Association between No/Lo alcohol product range and sales volume and neighbourhood income deprivation and urbanicity across retailers, 2018–2022. Intermediate models include neighbourhood income deprivation, urbanicity and standard alcohol sales. [file ADD-121-1863-s001.docx]

**SUPPLEMENTARY MATERIAL**

**Table S1**. Model diagnostics for associations between No/Lo alcohol product range, sales volume, and retailer-level predictors, 2018-2022. (Observations, n=28,346)

| **Model** | **Variables (adjust-ment)** | **Outcome** | **Type** | **AIC** | **BIC** | **R2** | **Log-Likely-hood** | **Mc-Fadden pseudo R2** | **Cox Snell pseudo R2** | **RMSE** | **Sigma** |
| --- | --- | --- | --- | --- | --- | --- | --- | --- | --- | --- | --- |
| Full adjusted Model (#3) (see Figure 3 in main text) | (IMD + UR + SAS) * Year | Product Range | Negbin | 48,769.35 | 49,025.10 | 0.30 | -24353 | 0.097 | 0.198 | 0.813 | 1.000 |
|  |  | Sales | ZIP | 117,800 | 118,300 | 0.68 | -58847 | 0.097 | 0.364 | 5.741 | 5.747 |
| Intermediate Model (#2) (see table S4 below) | IMD + UR + SAS | Product Range | Negbin | 50,742.87 | 50,800.63 | 0.19 | -25364 | 0.060 | 0.126 | 0.849 | 1.000 |
|  |  | Sales | ZIP | 122,500 | 122,600 | 0.54 | -61219 | 0.0607 | 0.246 | 5.926 | 5.927 |
| Intermediate Model (#1) (See table S3 below) | IMD + UR | Product Range | Negbin | 53,628.69 | 53,669.94 | 0.02 | -26809 | 0.00597 | 0.013 | 0.896 | 1.000 |
|  |  | Sales | ZIP | 130,000 | 130,100 | 0.06 | -64987 | 0.00293 | 0.014 | 6.162 | 6.163 |
| Univariate models (#0) (see table S2 below) | IMD | Product Range | Negbin | 53,636.30 | 53,669.31 | 0.02 | -26814 | 0.00579 | 0.013 | 0.896 | 1.000 |
|  |  | Sales | ZIP | 130,000 | 130,100 | 0.06 | -65018 | 0.00247 | 0.011 | 6.162 | 6.163 |
|  | UR | Product Range | Negbin | 53,938.4 | 53,963.21 | 0.01 | -26966 | 0.00015 | 0.001 | 0.900 | 1.000 |
|  |  | Sales | ZIP | 130,300 | 130,300 | 0.01 | -65136 | 0.00065 | 0.003 | 6.174 | 6.174 |
|  | SAS | Product Range | Negbin | 51,669.89 | 51,702.90 | 0.14 | -25831 | 0.04224 | 0.091 | 0.867 | 1.000 |
|  |  | Sales | ZIP | 124,100 | 124,100 | 0.48 | -62032 | 0.04875 | 0.201 | 5.998 | 5.998 |
|  | Country | Product Range | Negbin | 53,598.56 | 53,631.57 | 0.02 | -26795 | 0.00649 | 0.014 | 0.896 | 1.000 |
|  |  | Sales | ZIP | 129,800 | 129,800 | 0.08 | -64886 | 0.00447 | 0.021 | 6.158 | 6.158 |
|  | Year | Product Range | Negbin | 52,176.76 | 52,226.27 | 0.11 | -26082 | 0.03292 | 0.071 | 0.876 | 1.000 |
|  |  | Sales | ZIP | 126,500 | 126,600 | 0.35 | -63231 | 0.02987 | 0.130 | 6.064 | 6.065 |

Notes:

IMD = Income Multiple Deprivation; UR = Urban/Rural classification; SAS = Standard Alcohol Sales volume.

Negbin = Negative Binomial model; ZIP = Zero-Inflated Poisson model.

**Figure S1**. Mean weekly No/Lo alcohol product range by neighbourhood deprivation.

**
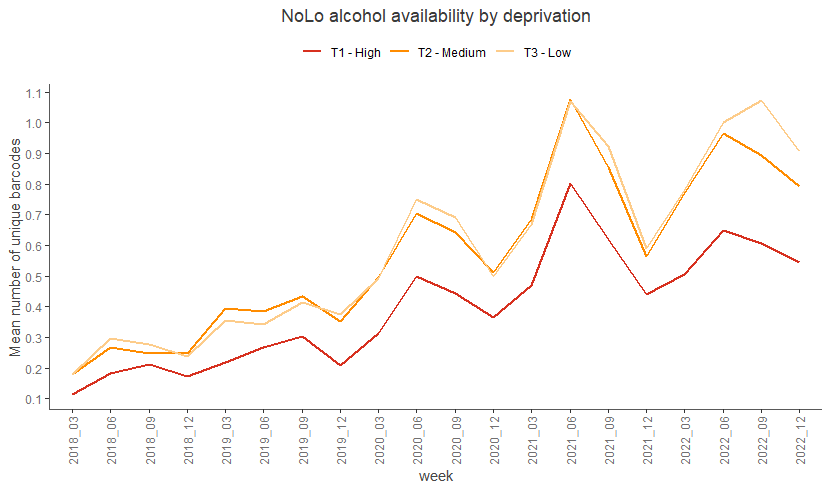
**

**Figure S2**. Mean weekly No/Lo alcohol sales volume (upper panel) and product range (bottom panel) by neighbourhood urbanicity.


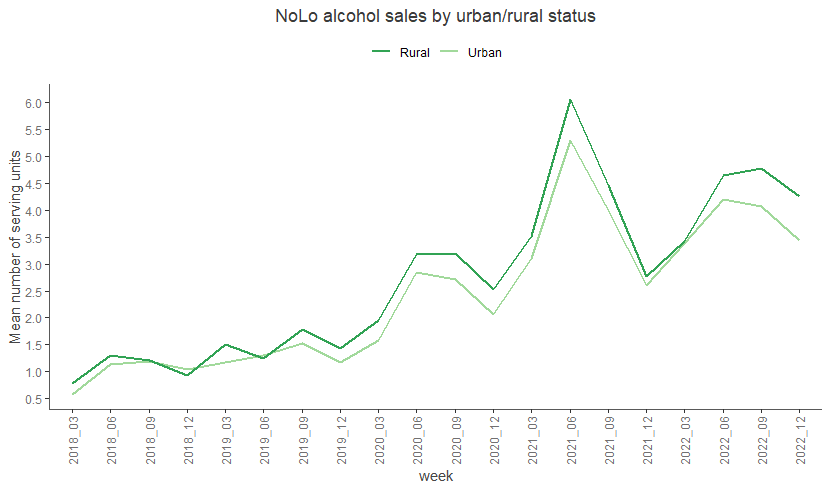


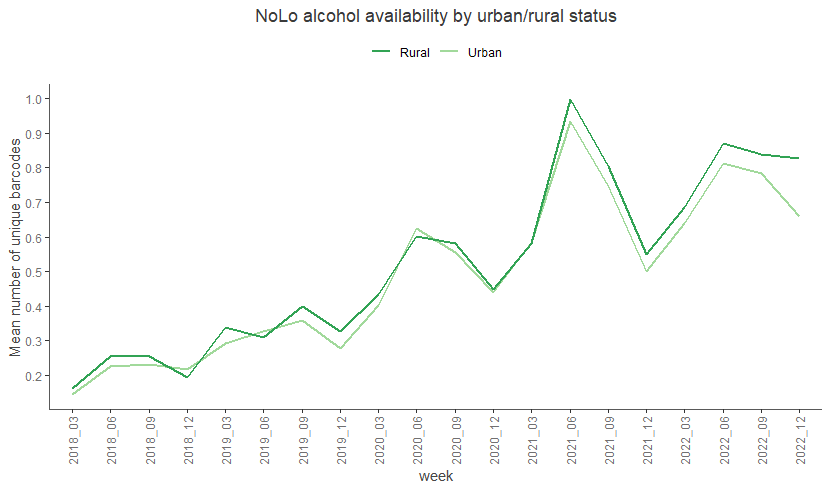


**Table S2**. Association between No/Lo alcohol product range and sales volume and neighbourhood income deprivation and urbanicity across retailers, 2018-2022. Univariate non adjusted models.

| **Predictor Domain** | **Predictor Class** | **No/Lo Product Range** | **No/Lo Sales** | |
| --- | --- | --- | --- | --- |
|  |  | No. of unique barcodes (count model)  IRR (95%CI) | Probability of no sales (Zero-inflation component)  OR (95%CI) | No. of serving units (Count component)  IRR (95%CI) |
| **INCOME DEPRIVATION** | IMD T1 | 1 | 1 | 1 |
|  | IMD T2 | 1.45 (1.38 - 1.52) | 0.67 (0.63 - 0.7) | 1.05 (1.03 - 1.07) |
|  | IMD T3 | 1.5 (1.42 - 1.59) | 0.63 (0.59 - 0.67) | 1.06 (1.04 - 1.08) |
| **URBANICITY** | Urban | 1 | 1 | 1 |
|  | Rural | 1.07 (1.02 - 1.12) | 0.93 (0.88 - 0.98) | 1.08 (1.06 - 1.1) |
| **COUNTRY** | England | 1 | 1 | 1 |
|  | Scotland | 0.67 (0.62 - 0.73) | 1.51 (1.38 - 1.65) | 0.81 (0.79 - 0.84) |
|  | Wales | 0.56 (0.52 - 0.60) | 1.72 (1.59 - 1.86) | 0.83 (0.81 - 0.86) |
| **STANDARD ALCOHOL SALES** | T1 - High | 1 | 1 | 1 |
|  | T2 | 0.55 (0.52 - 0.57) | 1.99 (1.87 - 2.11) | 0.66 (0.65 - 0.67 |
|  | T3 – Low | 0.28 (0.26 - 0.29) | 4.15 (3.88 - 4.44) | 0.51 (0.5 - 0.52) |
| **YEAR** | 2018 | 1 | 1 | 1 |
|  | 2019 | 1.55 (1.43 - 1.68) | 0.63 (0.58 - 0.7) | 0.92 (0.88 - 0.95) |
|  | 2020 | 2.44 (2.26 - 2.63) | 0.37 (0.33 - 0.4) | 1.14 (1.1 - 1.17) |
|  | 2021 | 3.37 (3.13 - 3.63) | 0.27 (0.24 - 0.29) | 1.51 (1.47 - 1.56) |
|  | 2022 | 3.58 (3.32 - 3.85) | 0.26 (0.23 - 0.28) | 1.49 (1.44 - 1.53) |

IMD T1 = most income deprivation category; IMD T3 = least income deprivation category.

**Table S3**. Association between No/Lo alcohol product range and sales volume and neighbourhood income deprivation and urbanicity across retailers, 2018-2022. Intermediate models include neighbourhood income deprivation and urbanicity.

| **Predictor Domain** | **Predictor Class** | **No/Lo Product Range** | **No/Lo Sales** | |
| --- | --- | --- | --- | --- |
|  |  | No. of unique barcodes (count model)  IRR (95%CI) | Probability of no sales (Zero-inflation component)  OR (95%CI) | No. of serving units (Count component)  IRR (95%CI) |
| **INCOME DEPRIVATION (IMD)** | IMD T1 | 1 | 1 | 1 |
|  | IMD T2 | 1.47 (1.40 - 1.55) | 0.62 (0.57 - 0.66) | 1.04 (0.98 - 1.11) |
|  | IMD T3 | 1.55 (1.46 - 1.64) | 0.57 (0.52 - 0.62) | 1.04 (0.97 - 1.12) |
| **URBANICITY** | Urban | 1 | 1 | 1 |
|  | Rural | 0.92 (0.88 - 0.97) | 1.14 (1.06 - 1.23) | 1.08 (1.01 - 1.15) |

 IMD T1 = most income deprivation category; IMD T3 = least income deprivation category.

**Table S4**. Association between No/Lo alcohol product range and sales volume and neighbourhood income deprivation and urbanicity across retailers, 2018-2022. Intermediate models include neighbourhood income deprivation, urbanicity and standard alcohol sales.

| **Predictor Domain** | **Predictor Class** | **No/Lo Product Range** | **No/Lo Sales** | |
| --- | --- | --- | --- | --- |
|  |  | No. of unique barcodes (count model)  IRR (95%CI) | Probability of no sales (Zero-inflation component)  OR (95%CI) | No. of unique barcodes (count component)  IRR (95%CI) |
| **INCOME DEPRIVATION (IMD)** | IMD T1 | 1 | 1 | 1 |
|  | IMD T2 | 1.66 (1.58 - 1.74) | 0.53 (0.5 - 0.56) | 1.09 (1.07 - 1.11) |
|  | IMD T3 | 2.09 (1.98 – 2.21) | 0.39 (0.36 - 0.42) | 1.2 (1.17 - 1.22) |
| **URBANICITY** | Urban | 1 | 1 | 1 |
|  | Rural | 1.11 (1.06 - 1.16) | 0.89 (0.83 - 0.94) | 1.16 (1.14 - 1.19) |
| **STANDARD ALCOHOL SALES** | T1 - High | 1 | 1 | 1 |
|  | T2 | 0.51 (0.48 - 0.53) | 2.22 (2.09 - 2.36) | 0.63 (0.62 - 0.64) |
|  | T3 – Low | 0.23 (0.21 - 0.24) | 5.65 (5.25 - 6.08) | 0.47 (0.46 - 0.48) |

IMD T1 = most income deprivation category; IMD T3 = least income deprivation category.

**Figure S3**. Association between No/Lo alcohol product range and sales volume and neighbourhood urban/rural status across retailers stratified by No/Lo alcohol product type, 2018-2022.

| **A) Beer** | | | | | |
| --- | --- | --- | --- | --- | --- |
| **No/Lo Product Range** | | **No/Lo Sales** | | | |
| No. unique barcodes | | Probability of no sales (zero-inflation component) | | No. serving units (count component) | |
| IRR (95% CI) | 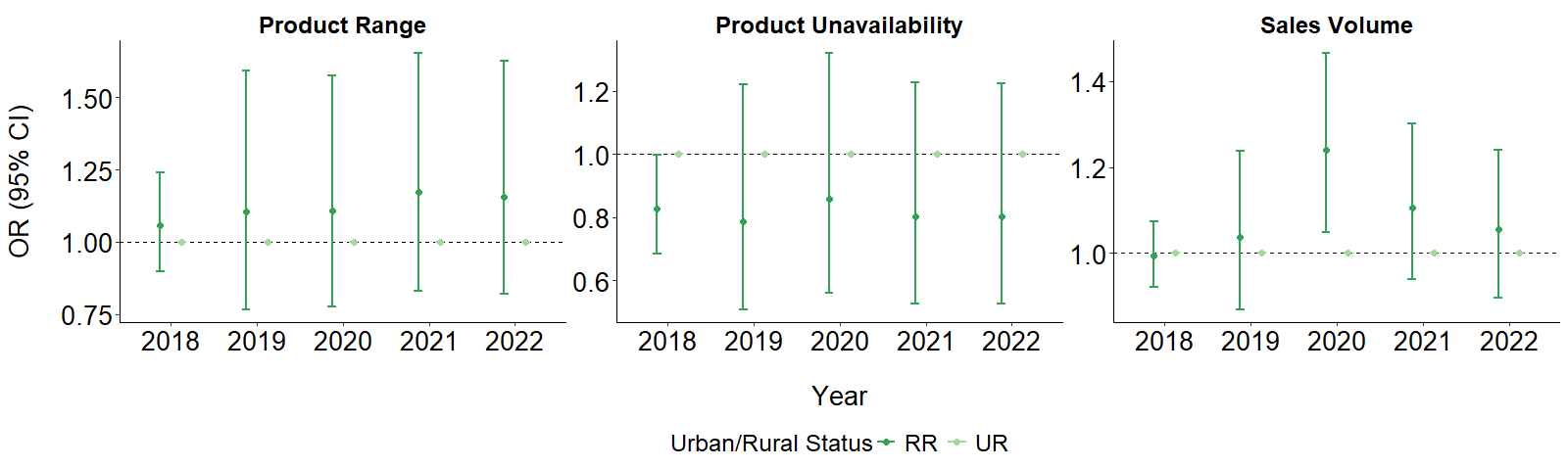 | OR (95% CI) | 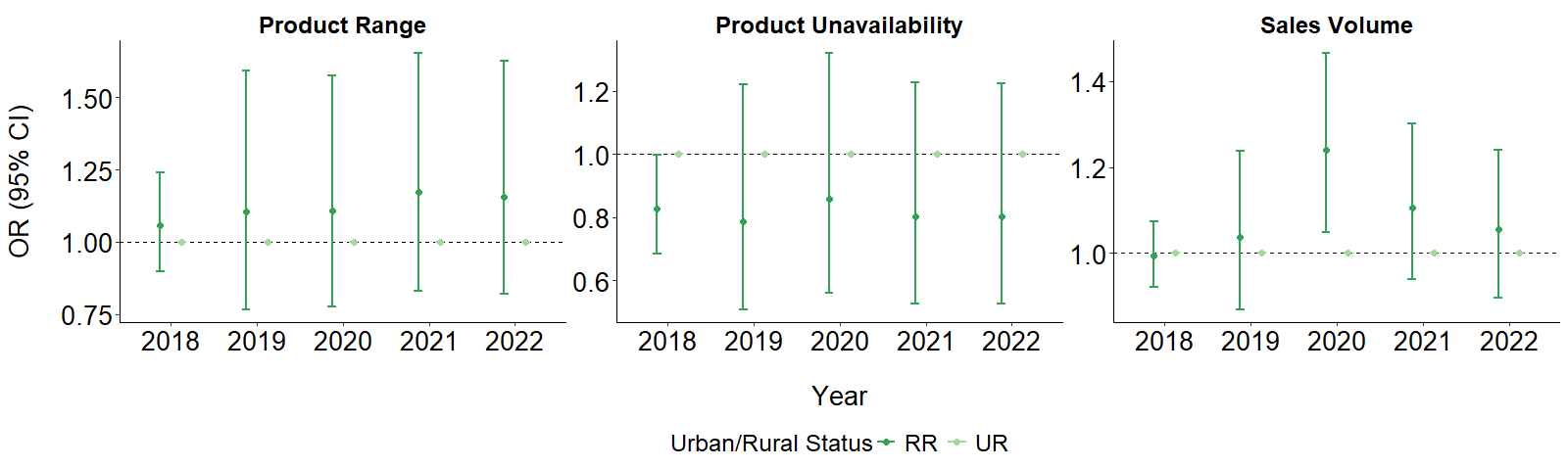 | IRR (95% CI) | 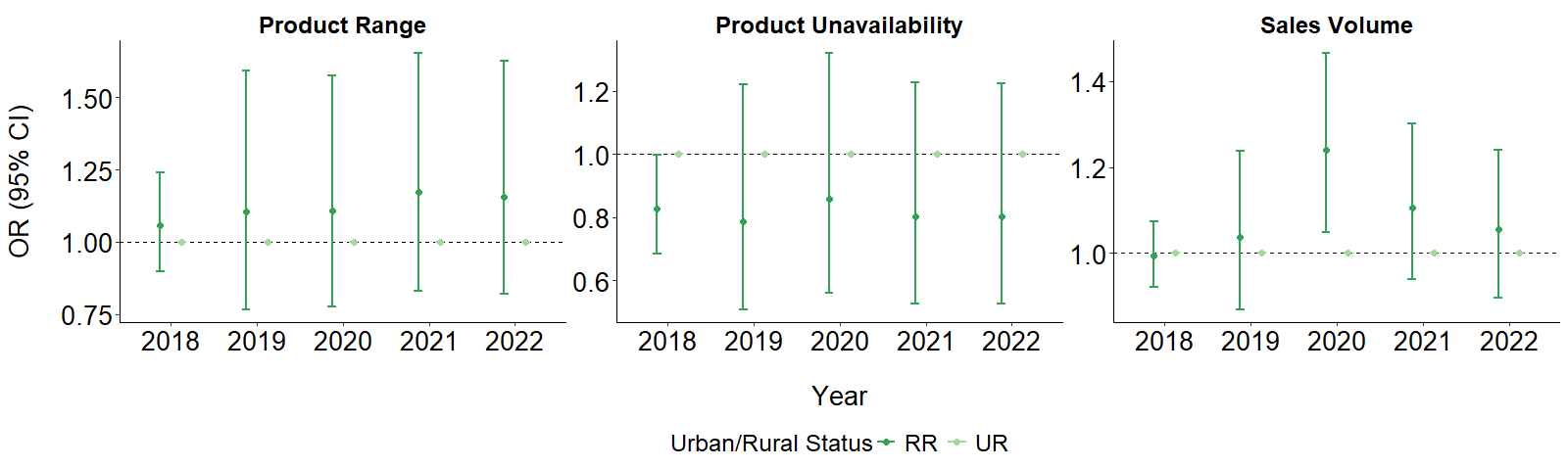 |
| **B) Cider** | | | | | |
| **No/Lo Product Range** | | **No/Lo Sales** | | | |
| No. unique barcodes | | Probability of no sales (zero-inflation component) | | No. serving units (count component) | |
| IRR (95% CI) | 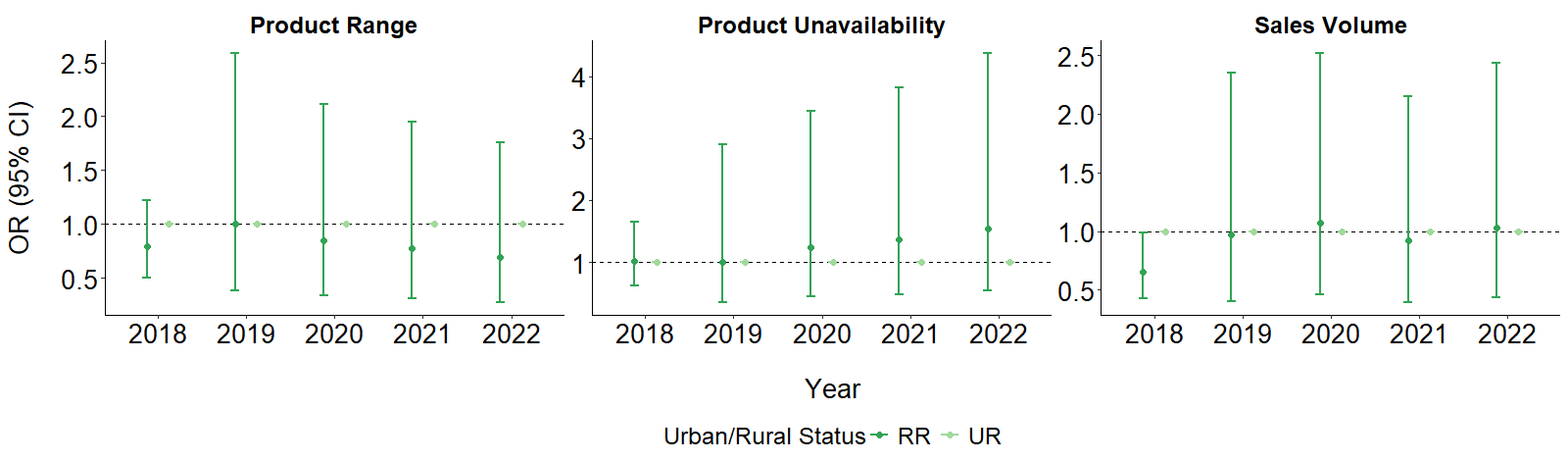 | OR (95% CI) | 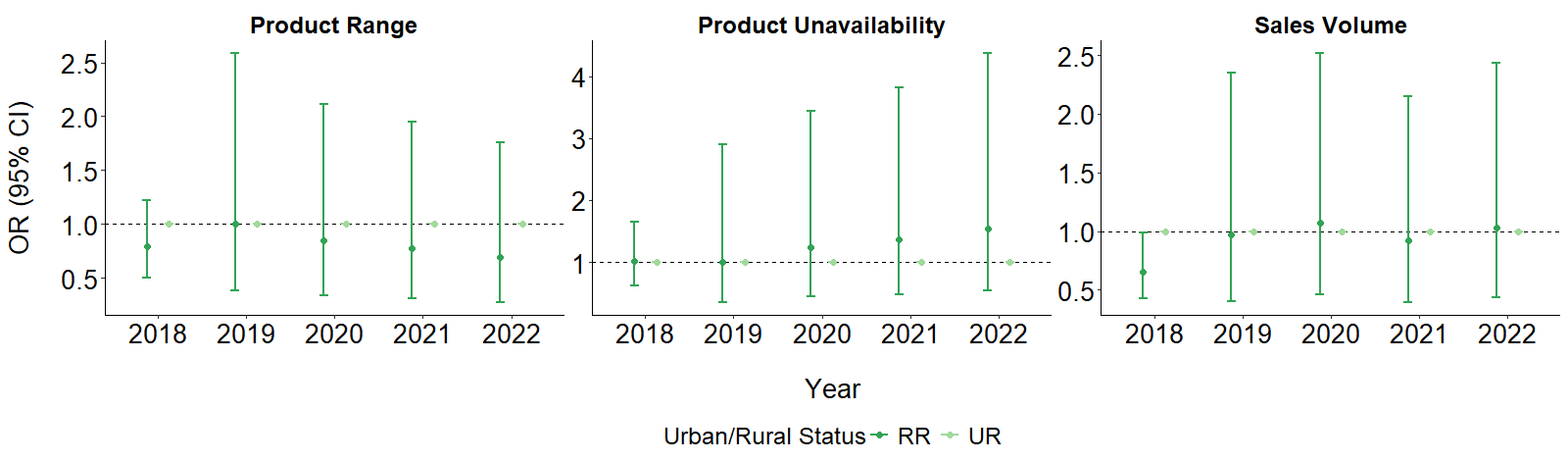 | IRR (95% CI) | 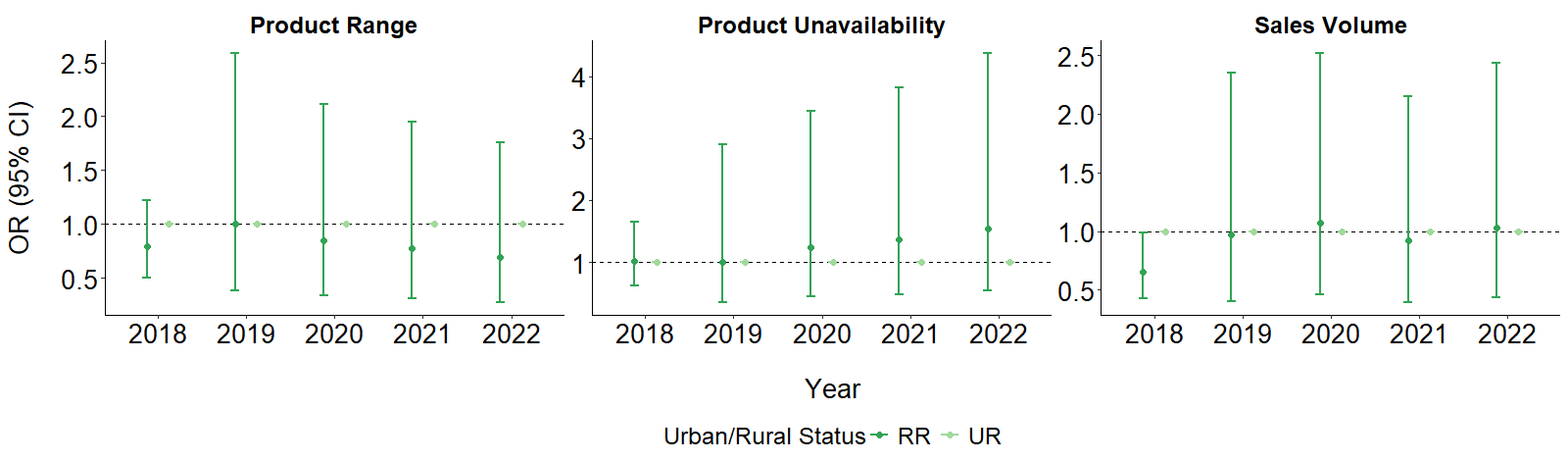 |
| **C) Wine** | | | | | |
| **No/Lo Product Range** | | **No/Lo Sales** | | | |
| No. unique barcodes | | Probability of no sales (zero-inflation component) | | No. serving units (count component) | |
| IRR (95% CI) | 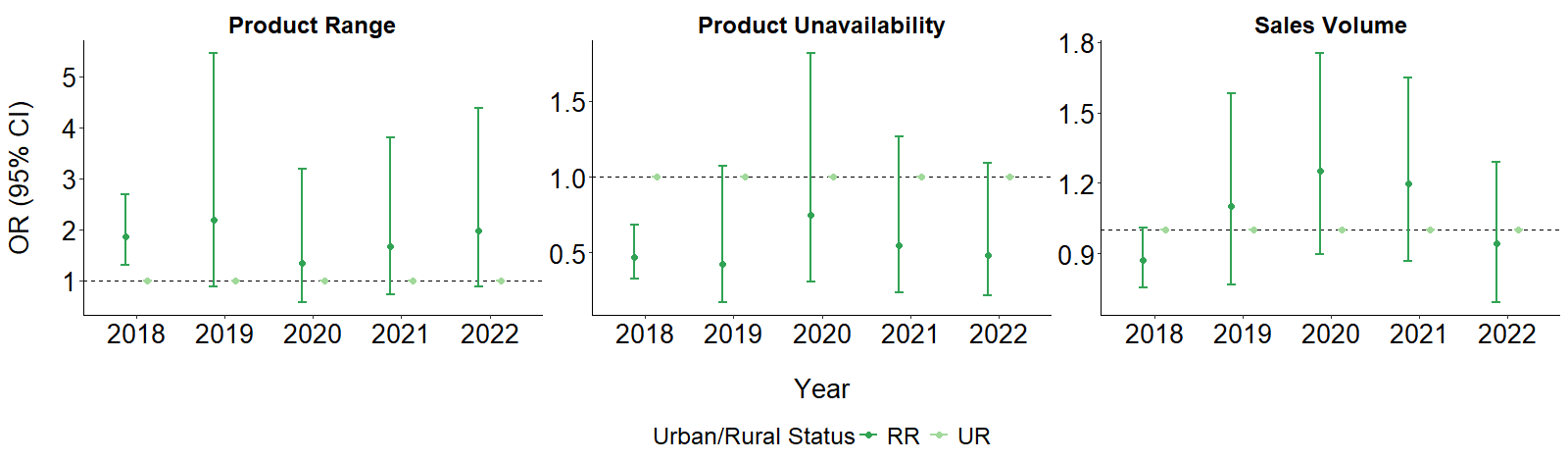 | OR (95% CI) | 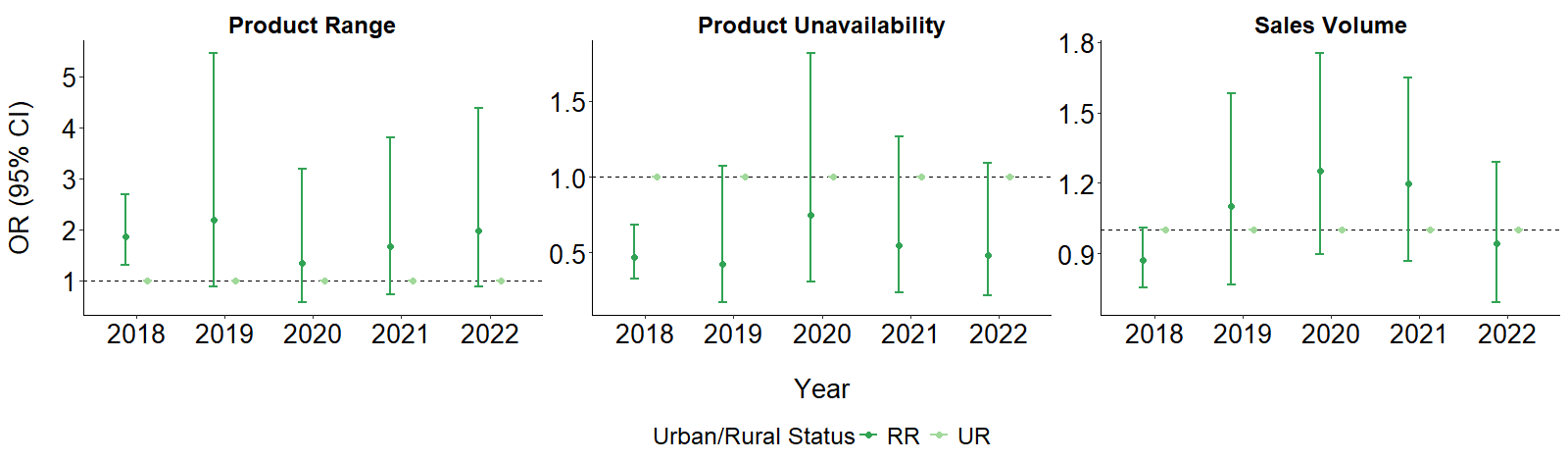  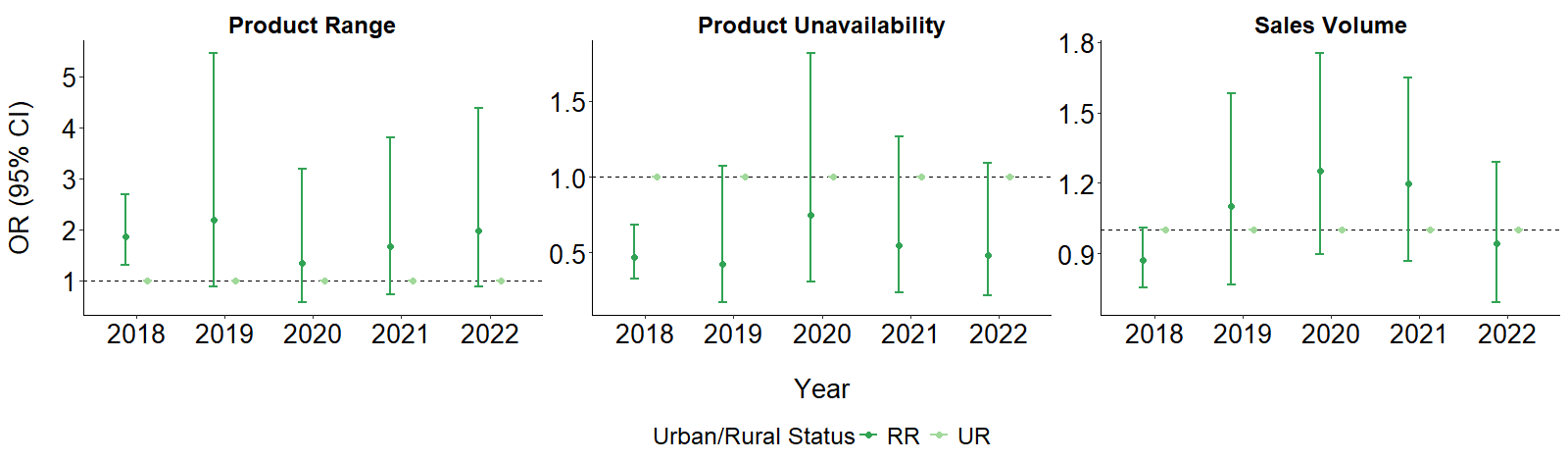 | IRR (95% CI) | 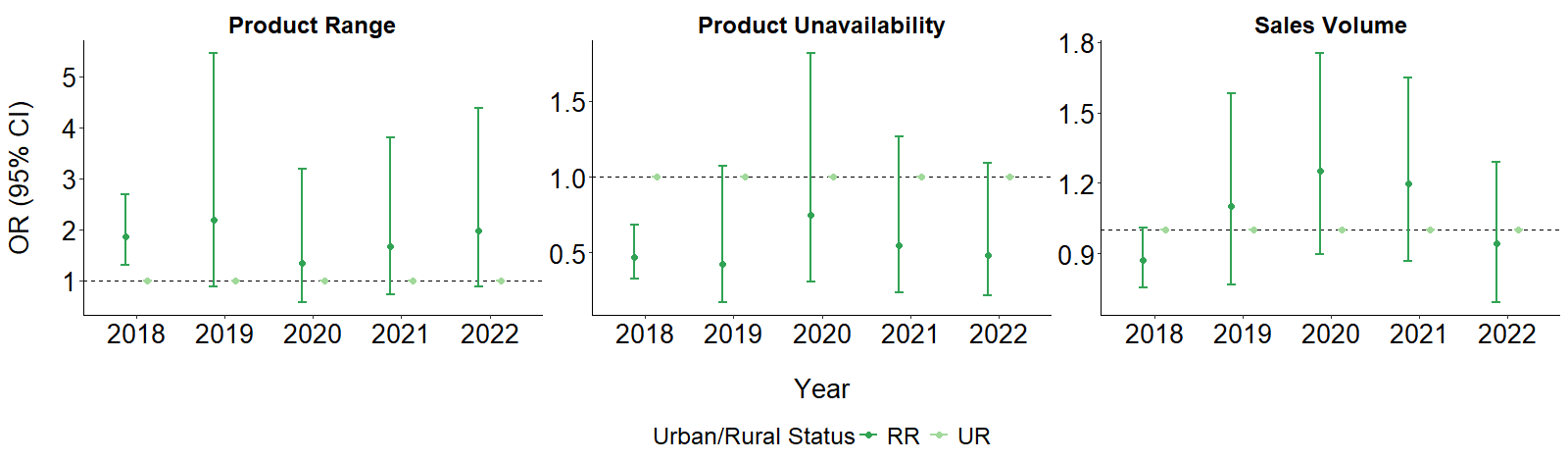 |
